# More that unites us than divides us? A qualitative study of integration of community health and social care services

**DOI:** 10.1186/s12875-020-01168-z

**Published:** 2020-05-29

**Authors:** Claire Mitchell, Abigail Tazzyman, Susan J. Howard, Damian Hodgson

**Affiliations:** 1 Alliance Manchester Business School, University of Manchester and NIHR Collaboration for Leadership in Applied Health Research and Care (CLAHRC) Greater Manchester, Booth Street West, Manchester, M15 6PB UK; 2 Salford Royal NHS Foundation Trust, University of Manchester and NIHR Collaboration for Leadership in Applied Health Research and Care (CLAHRC) Greater Manchester, Salford, UK

**Keywords:** Integration, Community services, Health care, Social care, Qualitative research

## Abstract

**Background:**

The integration of community health and social care services has been widely promoted nationally as a vital step to improve patient centred care, reduce costs, reduce admissions to hospital and facilitate timely and effective discharge from hospital. The complexities of integration raise questions about the practical challenges of integrating health and care given embedded professional and organisational boundaries in both sectors. We describe how an English city created a single, integrated care partnership, to integrate community health and social care services. This led to the development of 12 integrated neighbourhood teams, combining and co-locating professionals across three separate localities. The aim of this research is to identify the context and the factors enabling and hindering integration from a qualitative process evaluation.

**Methods:**

Twenty-four semi-structured interviews were conducted with equal numbers of health and social care staff at strategic and operational level. The data was subjected to thematic analysis.

**Results:**

We describe three key themes: 1) shared vision and leadership; 2) organisational factors; 3) professional workforce factors. We found a clarity of vision and purpose of integration throughout the partnership, but there were challenges related to the introduction of devolved leadership. There were widespread concerns that the specified outcome measures did not capture the complexities of integration. Organisational challenges included a lack of detail around clinical and service delivery planning, tensions around variable human resource practices and barriers to data sharing. A lack of understanding and trust meant professional workforce integration remained a key challenge, although integration was also seen as a potential solution to engender relationship building.

**Conclusions:**

Given the long-term national policy focus on integration this ambitious approach to integrate community health and social care has highlighted implications for leadership, organisational design and inter-professional working. Given the ethos of valuing the local assets of individuals and networks within the new partnership we found the integrated neighbourhood teams could all learn from each other. Many of the challenges of integration could benefit from embracing the inherent capabilities across the integrated neighbourhood teams and localities of this city.

## Background

Reorganising previously fragmented community health and social care services in England, whilst maintaining economic equilibrium, remains a challenge [1]. In common with health services across the world, the National Health Service (NHS) in England has, in recent years, seen an increasing focus on policies designed to integrate care between health sectors and between health and social care [2] with a greater emphasis on prevention and reducing health inequalities [3]. Historically social care was funded by government and provided by local authority councils and following reform it is now partly funded by local authorities, service users and their families with services provided by the private sector [4]. Integration of community health and care services continues to be promoted as a means to improve patient centred care, reduce costs, reduce admissions to hospital and facilitate timely and effective discharge from hospital [5–7]. This is in the context of a growing older population, who are living with complex multi-morbid conditions leading to greater health and social care needs [5]. The long history in this city of collaboration between the health services and local authorities has led to this being one of the first regions to reform health and social care towards an integrated care system.

Despite this policy focus, it remains the case that the integration of health and social care is not easy to define [8–10]. Integrated care is a widely used term and recent reviews have identified over 175 different definitions which range from outcomes-based to process-based [11]. There are some descriptions of ‘integration’ as a coordination of structure and processes, while ‘integrated care’ describes the experiences of the patient and their outcomes and highlights the confusion around terminology in the literature [10, 12]. Integration, both nationally and internationally, has to consider how integration is set up and this can range from organisations remaining separate but working in a co-ordinated way, to having a formal collaboration with the same governance or full integration where there is one single organisation [13]. Integration can involve different combinations of services [14] depending on whether it is focussing on specific conditions or wider general populations [15]. Integration can also be defined as outcome-based, measured through the impact on the individual receiving care, or process based, measured through the change to the system delivering care [16]. Integration is being implemented in various ways across the United Kingdom (UK), and while there has been some evaluation of this [17], there remains a paucity of research particularly around the operational challenges of integrated community health and social care services [18–20]. Few studies consider how the multiple experiences and perspectives of those delivering these services may affect this process [21].

Furthermore, the existing literature mainly reports on clinical integration where the institutions remain separate, with few examples of institutions collaborating or working in partnerships at organisational level [4]. The existing examples of clinical or professional integration have found increased patient satisfaction and a perception of better quality care and access [20]. However, the impact on other outcomes such as reducing costs, readmission to hospital and reduced length of hospital stay are less clear [20, 22–24]. Despite this mixed picture in terms of outcomes however, integration continues to be the prime focus in health and social care services organisation across the UK, with a combination of organisational, clinical and service integration considered essential to deliver better outcomes [7, 25].

This research intended to examine the context in this particular location with the establishment of a local care partnership working collaboratively to integrate and co-locate two main providers, community health and social care services, to form 12 community teams, four in each of the three localities that make up the city in question. Each community team will include nursing and social care professionals, as well as a team leader with or without a clinical background. The specific characteristics of this model of integration involved health and social care professionals co-locating and working together, as well as collaborating with GP and third sector partnerships, with the broader aims of improving wider population health and reducing health and social inequalities in this city of 500,000 people. At this point the system level of integration within the partnership was co-location, shared management, a partnership board, shared governance, combining some finances. This evaluation does not seek to measure the impact of integration, given the early stage of implementation, but instead examines some of the key factors encountered by those involved in integration at all levels of the partnership. This paper aims to report a contextualised understanding of the barriers and obstacles to this system level of integration, where community NHS and council services are brought together to work as one system.

### Contribution to the field

While other literature has reported mainly on partially integrated care services at team and service level there is a need for further research to continue to explore and report on the complexity involved in implementing system level integrated new models of community care services [20]. By interviewing the individuals involved at all levels, we develop an understanding of the multiple and sometimes conflicting perspectives to highlight factors perceived to be supporting or hindering integration.

## Methods

This study is a qualitative process evaluation based on semi-structured interviews. This process evaluation examined the way in which integration was implemented in the English city being investigated, seeking to identify challenges, enablers and adaptations made [26]. The interviews were conducted as the partnership was in place and the integrated neighbourhood teams were in the process of co-locating and working together. An interview schedule of broad questions was developed from a rapid literature scoping review. The schedule was designed to gain an understanding the context, barriers and enablers around clinical and system level integration from the interviewee’s perspective including impact on those receiving care and measuring progress of integration (Supplementary file 1). The sampling strategy combined purposive and snowball sampling to capture a diverse study sample. Purposive sampling was carried out to interview equal numbers from community health (employed by the NHS) and social care (employed by local authority). At the operational level community health professionals all had nursing backgrounds and social care staff were all social workers. Strategic level staff were either employed by the NHS or local authority council. We ensured equal representation of health and social care professionals across the three localities.. From initial interviews snowball sampling was used to identify further participants. At an operational level we recruited respondents with a variety of experience and grades to give a broad sample of participants. In total 24 interviews were carried out (Table 1).

**Table 1:** Final study sample across all schemes

Participant role and background	Interviewees (social care)	Interviewees (health care)	Total
Strategic level leads	3	3	**6**
Operational staff: Area 1	3	4	**7**
Operational staff: Area 2	3	3	**6**
Operational staff: Area 3	3	2	**5**
**Total**	**12**	**12**	**24**

All semi-structured interviews were carried out in person by either one or two interviewers (conducted by authors 1, 2 and 4) at the individuals’ place of work between April 2018 and November 2018. The interviews lasted between 45 min and 1 h 30 min, with most being approximately 1 h long. The interviews were all audio-recorded, with participants’ written consent, transcribed verbatim and anonymised before being transferred to NVivo11 software to store and manage the data [27]. Field notes were made during and after interviews.

Data analysis followed a thematic approach and was carried out between October 2018, concurrently with the final interviews, to December 2018 by authors 1, 2 and 4. A coding framework was created by the team based on the findings of our literature review and other key case studies and reviews [9, 28]. This was focussed around six key themes each covering an area of activity undertaken during the implementation of integration. These were: 1) clinical, 2) informational, 3) organisational, 4) financial, 5) administrative and 6) normative. In addition further codes were added to the framework inductively as appropriate and then coded across all transcripts [29, 30]. The coding process was iterative, conducted by authors 1 and 2, with the framework refined by consensus of the whole team. Following this initial coding findings were then compared across all three localities as well as between operational and strategic participants to establish similarities and differences across them. The agreed coding framework was applied to the complete dataset by authors 1 and 2, co-coding throughout.

## Results

In this paper we aimed to provide an understanding of the context, enablers and barriers to the process of integrating two previously separate entities into one partnership, considering the system level and care delivery as described by the professionals involved. In this paper we report in detail three key elements (taken from the original 6 themes) from our thematic analysis: 1) the shared vision and leadership of integration; 2) organisational level integration; 3) professional workforce integration. This paper discusses each of these and provides recommendations for integrated teams.

### Vision and leadership

A positive vision of integration was expressed by all interviewees, across health and social care and the three localities. At both strategic and operational levels similar language was used in depicting this vision. This suggests that the vision of integration has been clearly communicated to all staff with evidence of buy-in across the board, from leadership through to operational level. The importance of integrating teams was described in terms of perceived impact on those receiving care, with a consistently positive picture presented by interviewees:

*”... Even if you did need to see five different people, it should feel like the same service. And they should all know why they’re there and know about the other person and all have the same goal.” (Strategic social care, interviewee 3)*

Staff described how integration should lead to more seamless care, one professional would be key to coordinating care for an individual, ensuring people were not referred onto different services where they could languish on waiting lists but would get access to the relevant professional when needed. There was also a belief that individuals needing care would be less likely to have to repeat their personal situation and history multiple times to each new professional and that co-ordination would mean the different professionals would know who was visiting and when, so individuals were not overwhelmed by visits on the same day.

The impact of health and social care professionals working together in a closer way was described positively and seen by the majority of interviewees as one of the great benefits of integrated working. Despite the challenges of working closely, interviewees described the potential benefits of joint working, closer collaboration and a deeper understanding of each other’s roles:

*“…and I would’ve actually said, I haven’t got a clue. I haven’t got a clue. I don’t deal with that. But now because I’ve worked with a...and I’ve been out and I’ve assessed a patient with a social worker I can say to them, you know, there’s different levels of care.…..So I can discuss it.” (Operational health area 2, interviewee health b)*

Staff reported that having an understanding of each other’s roles from joint working and integration generally would support the seamless care described above. The different professionals felt that they would be able to give more information to the patient about what other services could provide and this knowledge would help them find out more pertinent information and to bring in other professionals at the most appropriate time point as discussion would be much quicker and easier to do when co-located.

Leadership of the new partnership, while embedding a positive vision, was described as needing to have courage and a willingness to take risks as integrating health and social care represents, in many ways, a move into uncharted territory creating a more permissive environment:

*“You need to have the right leadership, the right vision, with a real kind of… you have to be brave, senior leaders have to be brave.” (Strategic social care, interviewee 2)*

It was recognised that ‘brave’ leadership was challenging, particularly when integration is under such close public scrutiny. Although the integrated partnership is fostering distributed leadership, we found that those interviewed thought that leaders tended to revert to tried and trusted ways of working and with colleagues they knew and trusted.

Strategic level staff described the vision for distributed and devolved leadership and expressed the need for highest level leaders of the partnership to do things differently, particularly around budgets and delegating responsibility. There was a sense from the strategic level staff that there should not be a standard approach to devolving leadership and this would need to be flexible due to the differences between the various teams in terms of progress towards integration, variation in staff experience, skills and knowledge, as well as the inherent differences between localities and within these neighbourhoods across the city. However, some reported that devolved leadership was circumscribed as staff were not given sufficient authority for local decision making or that staff were not aware of this aspiration. Several perceived a disconnect between what leaders in the partnership said about distributed leadership and the implementation of it:

*“So, people need to understand that they are empowered to do that* [31] *and that the solutions will come from the staff not from a small bit of the organisation, less than one per cent who would be seen as decision makers. This is decision-making from the ground up.” (Operational health, area 1, interviewee a)*

Our interviews with both operational and strategic staff suggested that safe transfer of care for all those using health and social care services to the new partnership, individuals not being lost in the system or breakdown in delivery of care services, has been of paramount importance to the leaders. In some interviews, however, staff felt that excessive concern around safety, particularly at a leadership level, was hindering the development of different or innovative ways of working and delivering care:

“*…we’ve got to keep people safe. So, that work has to be done. But there’s also, if we just do that stuff, and we keep on doing that stuff, actually things are not going to get any better, people are still going to die, people are still going to have long term conditions, A&E will still be busting. So, there is something about, how do we change the way we do things?*” *(Strategic social care, interviewee 1)*

There was a broad recognition from all interviewees that the vision of integration was not reflected in the prescribed measures of success for integration. Generally, nationally and locally agreed measures are reductions in hospital admissions and length of hospital stay. Many people interviewed felt that these measures were seen as important by senior leadership but interviewees personally considered them to be poor measures of success of the integrated neighbourhood teams. Several observed that the focus of integration was on prevention (by enabling individuals to take responsibility for their health, by signposting other services or by reducing impact on other services) and were unconvinced success in this regard could be identified in the short to medium term:

“*…aims are reducing A&E admissions and stays in hospital and stuff. But the bigger thing is, we will talk about improving health outcomes, enabling people to live longer… Well, in terms of the prevention work, and some of the stuff that we’re doing, there’ll be no deliverables [affecting hospital admissions/stays]…” (Strategic social care, interviewee 1)*

There was a sense that the demand from a growing, ageing population and corresponding health and social care needs cannot be solved by integration alone, as demand will continue to rise. There were many comments therefore that integration should be judged against meaningful measures. The view of the individual receiving the care was suggested as a possible meaningful measure by some, the quality of the integrated services and the impact of integration on staff performance was also suggested:

*“One way that I would consider to measure it would be to see how effectively the teams are working, how efficiently the teams are working. So if the teams are struggling and have unallocated cases, huge backlogs, people off sick, people unable to go out and to kind of do the job because they’ve got to stay in the office for whatever reason. So if those are happening, that’s certainly an indicator that things aren’t working.” (Operational social care, area 2, interviewee b)*

### Organisational level integration

Organisational factors influencing integration were raised at all levels by health and social care staff from the clinical delivery of care to differences in human resource practices. The issue raised most frequently related to a perceived lack of operational detail in integration planning, a concern felt particularly by those working on the frontline. While staff reported a clear broad vision for integration, the majority reported a concern at the lack of detailed planning or a lack of communication of this throughout the partnership, highlighting the challenge of translating principle into operational detail:

*“That kind of reflects the situation really, that there are kind of big gaps and uncertainties, and also, probably, a lack of cascading messages down and a lack of kind of information that’s...you know, we know the headline that we’re leaving and things are happening, but I think a lot of the detail is lost and not fed down always.” (Operational social care, area 2, social care b)*

Many staff reported a key aim of integrating services was to have clear, streamlined care pathways for those needing either, or both, health and social care. The two main benefits anticipated were: a clear single point of entry and a seamless system to involve other health or social care professionals as appropriate without people getting lost in the system or being repeatedly referred on to alternative waiting lists for other health and care services. Although the detail of how this will work in each neighbourhood team was still to be finalised, staff clearly recognised how this could improve the experience of those accessing services:

“*So that whole thing around, we’re supposed to be integrated, so the fact that, I’ve got a big bugbear about referrals, so that idea that somebody has to be referred here, and then they have to be referred there, and nothing is ever joined up.….*” *(Strategic social care, interviewee 1)*

There was an awareness of the complexities involved in bringing together two organisations. This was particularly apparent when discussing human resource policies and practices where health and social care professionals have different grading, pay and responsibilities. Staff clearly had concerns that working closely together where there was not parity of grading and responsibility for example could lead to animosity between team members:

*“So, what I would say is, we are trying to bring the services together, to integrate them, and that will take some teasing out, because they all have different budgets, different management structures, different professional bodies. They have different training and development needs, they all have different policies, different procedures.” (Operational health, area 3, interview a)*

Inadequate information systems were raised as problematic by every interviewee, specifically the use of different IT systems for human resource and clinical work across professions and organisations. Staff were restricted in what clinical data they had access to and this is considered a barrier to streamlining working practices. The lack of a joined up clinical IT system across community services was identified as having a negative impact on data sharing when health, social care and services they collaborated with including GPs, mental health services and emergency responders all held different information pertaining to individuals with health and social care needs. At an individual level, there seemed to be widespread concerns around what information could be shared and who it can be shared with. This concern around data protection related to a perceived lack of trust between services; from acute to community, and between health and social care. This lack of coherence about who could access what information was understood to be a potential risk to individuals and a safeguarding issue. Data sharing could improve staff safety; for example if all staff were fully informed about dangerous social circumstances, they could make appropriate risk assessments when lone-working and carrying out home visits. Sharing complete data about individuals could also reduce unnecessary referrals to other services:

“*Like I rung the hospital yesterday and asked for a copy of somebody’s capacity assessment and the discharge facilitator said to me, she was like, oh, I don’t know if I can send you that because of confidentiality. I was like I can’t make the decisions that I need to make ...” (Operational social care, area 2, interviewee c)*

There was a perception that improved relationships between services and professionals could improve trust which would give more confidence around data sharing. This was seen to be a potential benefit of integration, where staff understood the limitations of information systems and accepted that seamlessly streamlined technical tools across all services and professions were an unlikely development in the near future.

Co-location, a key element to this integrated care service, was reported by the majority of people to be a necessary aspect of integration. Many felt physical co-location would be a way of facilitating integration and fostering trust, relationships and shared working. A possible benefit may also be around greater confidence in data sharing:

*“…co-locating, sharing the same building together, and in order for me to have district nurses information, or in order for me to have information from the GP if I am in the same place as them, and they know that…yes, this is way forward, part of integration, I think, that would make it very easy.” (Operational social care, area 3, interviewee c)*

Several, however, felt that co-location was not sufficient on its own without investment and support in integration and shared locations did not automatically lead to integrated professional teams:

*“… because putting a bunch of people in a room together, doesn’t mean that they like each other, that they’ll work together, or that they’ll be any more efficient than they already are.” (Operational health, area 3, interviewee a)*

### Professional workforce integration

Overwhelmingly, both the health and social care professionals interviewed expressed the same concerns and anxieties regarding their professional identity and boundaries. Both health and social care interviewees reported that they believed the other professional group did not fully understand their professional responsibilities, duties and governance. Governance within health care was considered to be to the relevant professional body (such as The Nursing and Midwifery Council), clinical guidance and the NHS, whereas social care was to the professional body (Health and Care Professions Council), local and national government and the law. This perception of core differences between the health and social care services is perhaps best highlighted by the ongoing confusion reported by several interviewees around terminology of what to call people using services. Both health and social care staff at strategic and operational level indicated they were unsure whether to call individuals receiving care “patients” or “citizens” and that this seemingly basic terminology issue could act as a barrier to communication and highlighted the perception that the detail of integration had yet to be decided.

The majority of comments reflected tensions and lack of understanding or trust between health and social care, but issues also arose within health between different professional groups such as district nurses and active case managers (also nurses), and between community and acute services. There was a perception from social care staff that they were dominated by the much bigger NHS:

“…*it feels like it’s so hospital-centric, the whole system, you’re either in hospital or out of hospital services. People have short episodes of their lives hopefully in hospitals, then they live in their own homes, in neighbourhoods.*” *(Strategic social care, interviewee 2)*

While social care felt overshadowed by the bigger health sector, both health and social care staff working in the community reported feeling neglected compared to acute care services. Acute care was considered by many to be seen as more important, to be better resourced, to have better access to information and to lack an understanding of what community care entailed. Community staff reported their concerns about individuals being discharged without sufficient attention to the handover of care leading to significant issues for community staff to pick up the pieces in difficult circumstances:

“*It’s a massive barrier. It really is a big barrier and it’s a shame. Because if we all came together, the hospital and us, it’d just make things so much easier, and that ride will be so much more bearable.*” *(Operational health, area 2, interviewee C)*

Social care staff felt there were fundamental differences to health staff, related to their understanding and implementation of the mental capacity act. There was a suggestion during the interviews that social care staff were more comfortable than health professionals in accepting people making ‘unwise’ decisions and more comfortable with higher levels of risk compared to health staff. All these ‘differences’ were considered in the interviews as potential barriers to shared responsibility and trust:

“*We do have very different kind of ideologies, and really my experience is that the health professionals do tend to be [more] risk-averse.*” *(Operational social care, area 2, interviewee b)*

Health care staff had their own concerns around responsibility, largely relating to their professional accountability and duty of care to people receiving health and social care services, accountability it was felt that other professions may not be aware of. Health professionals reported a sense of great responsibility towards those who come under their care, due to the nature of offering 24 h care or due to their professional standards. This generally made health staff feel as though both social care and acute health services might offload responsibility for certain tasks to them:

“*So then, what usually happens, is the district nurses pick it up, because they think, well somebody’s got to do it, and we have a duty of care, and nurses feel, as part of their professional registration, that they have a duty of care….*” *(Operational health, area 3, interviewee a)*

Both health and social care staff explained the complexity of ‘trusting others’ in light of their differing professional responsibilities, with the concern that the different ways in which other professionals work may leave them professionally accountable in the case of incidents:

“*Well I think the first thing is that we have statutory responsibilities. So, I think it's a big learning curve for our health colleagues to understand the importance of that, that we are guided by legal requirements, we're not just doing it because somebody thought it was a good idea that somebody should have a care package.*” *(Operational social care, area 2, interviewee a)*

Both health and social care staff were concerned about being managed by people from different professional backgrounds who may not be familiar with their professional codes of practice and current evidence base. There were many reports of positive experiences of inter-professional working and it was anticipated by many of the interviewees that increasing inter-professional collaboration would have benefits for both staff and individuals receiving care. There were also many suggestions around the need to share knowledge, to educate others about roles and to engage in joint learning and development alongside other professional groups. The ability to give further information about health or social care when from a different professional background was seen by some staff as a great benefit to the individual and their family, as well as the ability to know when another professional would have something to offer.

A key point raised, particularly by operational health staff, was the importance of GPs being involved in the integrated teams including meetings. GP involvement was seen as a real potential benefit to integration and staff described how engaging with GPs about particular individuals could be really difficult. It was suggested by other professions that GPs may view involvement in integration as contributing to an increasing and already unmanageable workload:

“*…for us working in the community the GP is at the heart of everything. And if a GP is not part of your integrated team, what do you call integrated? I think it needs to be looked at, where does the GP fit within this integrated working?” (Operational health, area 1, interviewee d)*

Convincing GPs that involvement in the integrated teams and participation in team meetings and gaining buy-in was considered essential to delivering seamless integrated care.

## Discussion

This study has explored the context, enablers and barriers to integrated health and social care teams in community services from the early development of a single integrated partnership where previously separate entities from NHS and social care work together as one entity. Our analysis identified three key influential factors that may affect the success of integrated health and social care: the vision and leadership of integration; organisational level integration and professional workforce integration. This evaluation draws on the insights of staff across localities, health and social care, at both a strategic and operational level, and constitutes a broad considered overview of how integration is understood among those most directly involved, highlighting factors which support or hinder the integration of health and social care. When this evaluation was carried out (April 2018 to November 2018), although the partnership was operating as a single entity, the 12 integrated neighbourhood teams were at slightly different stages but on the cusp of integration. The move to develop the 12 integrated teams was an ongoing process, in some teams the lead roles had been advertised and co-location was imminent, while other teams were still at a planning stage.. The opportunity to interview staff at this key point in the process has offered some useful context, insight and recommendations for this ongoing iterative process, with some important learning for others embarking on this transformational change. Integration, while high on the national agenda, has mixed reports of impact in the literature [20]. Integration at any level is widely acknowledged as a hugely challenging and complex undertaking [21] and this paper contributes to our knowledge.

The positive vision of integration expressed at all levels in our interviews is an important starting point and achievement for the partnership. The literature reports that integration can be hard to define, [16, 32] and for transformation plans to be successful there needs to be a clarity of vision and purpose to overcome organisational and professional barriers [33]. In terms of leadership there remained a feeling that broadly those leading the organisation were not delegating leadership responsibility although this may be due to poor communication or a lack of understanding of distributed leadership. Recommendations include continuing to embed the vision of integration and the role of the neighbourhood teams within the partnership particularly with new staff, to encourage senior leaders to work with operational staff to support local decision making and to foster the ethos of distributed leadership.

Although we found this coherent vision of integration and a shared clarity on how it could improve delivery of health and care services, there was a widespread perception that this was not being effectively evaluated by the official measures of success (reducing hospital admissions and length of hospital stay). The literature suggests defining what is being measured is difficult in itself, and establishing how best to measure this, including success/failure, is yet more difficult [32, 34, 35]. Potentially this raises challenges, where there is a perception that national and regional policy is often wedded to measure impacts through specific acute hospital figures which may be difficult to link to the prevention agenda of integration. This can reinforce feelings that acute care is prioritised at the expense of out-of-hospital care and the wider prevention agenda in the community. There is potential value in exploring preventative work, self-care and improved quality of service delivery when developing impact evaluation of integrated neighbourhood teams. We recommend integrated neighbourhood teams are actively involved in developing meaningful measures based on robust logic models of integration to demonstrate the intended benefit of integrated working.

The evidence suggests that the key challenge to integration of teams, services or organisations lies at the system level [16, 32]. The challenges encompass issues such as finances, resources, workforce capacity and capability [32]. This study looked at system level integration, designed to effect change at multiple levels; process (changing way services are delivered), service level (quality of care and changing resource use) as well as system wide impact level (changing use of primary care and community service). A lack of coherent, integrated technical information systems covering health and social care has previously been found to hinder integration [36], and was flagged by the majority of our interviewees as a barrier. Sharing information and data at an individual level is also known to be affected by the historic division of health and social care, [37] which is affected in turn by trust and wider underpinning relationships. The long-standing separation of the health and social care systems hinders integration as separate human resource departments and arrangements exist, with inconsistent grading, terms and conditions and responsibility structures [38]. Ongoing plans to co-locate the integrated neighbourhood teams are recommended to support data sharing and further streamlining of human resources. This paper will be of value to those developing integration of health and social care from a system level, and adds to the much needed evidence base of the early stages of integration where we have existing institutions working in partnership to provide integrated health and social care services.

We know from the evidence that a lack of understanding between different professions can hinder integration and that this lack of understanding can lead to conflict within teams affecting shared decision making and communication between team members [39, 40]. Although there is research on integration in healthcare and research around professional identities in healthcare generally, there is limited research around professional identity in the rapidly evolving world of integration [41]. There is some evidence that suggests subconscious bias of professionals involved in integration can influence behaviours to hinder integration, and challenges to the status quo can be destabilising for people [21]. Investment in shared learning and development initiatives across professions, teams and localities could improve working relations as people develop a richer understanding of each other’s responsibilities and governance.

The NHS Long term plan outlines the importance of dissolving barriers and their vision of seamless integration includes GP involvement in integrated teams [7]. A crucial issue that was raised by many of the interviewees was the importance of having GPs actively involved in the integrated teams. Many suggest it is important that GP involvement does not lead to a more medicalised model affecting the person-centred aims of the integrated teams [40, 42]. It is clear that further development work by senior partnership and GP leaders for sustainable and resourced involvement in integration would support the development of the integrated neighbourhood teams.

The recent publication of the NHS long term plan [7] and the final report of the Lord Darzi Review [43] as well as existing integration research [24, 28], all continue to highlight some of the key themes we report on in this paper. Specifically the need to investment in; wider population health not just healthcare, digital infrastructure across health and care, social care funding, staff delivering health and social care, resources to transform health and care acknowledging the time this takes [43]. The long term vision for the NHS clearly highlights that integration is a key focus as integrated care systems take priority leading to autonomy of budgets and performance [7, 24]. Integration of health and social care partnership and the development of integrated neighbourhood teams in this setting has shown us the challenges of putting such aspirations into practice and recommendations to learn from the barriers to integration. Integration continues to be a driver and the focus for the NHS throughout England [7] and although there is huge variety in context and implementation those involved must benefit from and consider the growing body of evidence related to integration of community health and social care [20]. There is an acknowledgement of the time taken to transform health and social care [44] and an understanding of the need for partnership working rather than competition, as well as a recognition that the focus of integration should be around improving health and care rather than balancing the books of the NHS [45].

### Highlight limitations of the study

We accessed the experiences of people working at strategic and operational levels within the integrated partnership but recognise the views of these 24 participants are in a specific location and context. This study is not an outcome evaluation of the integrated partnership, nor is it an evaluation of the integrated teams, but provides a rich contextual view, at a specific time point, of teams moving towards integrated health and social care delivery. This study did not capture service user experiences or views; this is a limitation of the current study and would be of interest in future research.

## Conclusions

Integration is complex and challenging at organisation, professional and service level. The vision of integration has real clarity throughout this new partnership to its benefit, but the barriers to achieving this are tangible at all levels, across all professional groups and across the localities. Key recommendations are the need for greater dialogue between leadership and key partners and more joint-working and shared learning particularly across professions, teams and localities to address the deeper tensions arising from professional specialisms. It is perhaps appropriate that many of the solutions entail capitalising on untapped potential through the partnership and sharing knowledge to overcome challenges. Given the widespread commitment to the vision of integration there is clearly more to unite health and social care than divide them.

## Supplementary information

**Additional file 1.** Interview schedule for integration of health and social care

## Data Availability

Consent was not obtained to share data with other researchers outside of the immediate research team due to maintaining anonymity of data.

## Abbreviations

NIHR CLAHRC GM: National Institute for Health Research Collaboration for Leadership in Applied Health Research and Care (CLAHRC) Greater Manchester
UK: United Kingdom
GP: General practitioner
NHS: National Health Service
