# Supplementary material for: More that unites us than divides us? A qualitative study of integration of community health and social care services
Source: BMC Fam Pract. 2020 May 29;21:96. doi: 10.1186/s12875-020-01168-z (PMC7260839; doi:10.1186/s12875-020-01168-z)
Supplement: Supplementary file 1 — Additional file 1. Interview schedule for integration of health and social care [file 12875_2020_1168_MOESM1_ESM.docx]

**Interview schedule for integration of health and social care:**

1. To introduce project aims, purpose of interview, agree anonymity and sign consent
2. Could you describe your current role and role within the integration
3. Integration definition
   - - What does the partnership mean by integration?
     - What does it mean to you?
     - What difference do you think it will make?
     - How will you define whether a team is integrated or not?
4. How will integration affect the health and social care needs of local population?
   - - What will this offer them?
     - How will it be different to before?
     - Is this a top down or bottom up approach?
5. What organisations are involved?
   - - What professions?
     - Members of the MDT?
6. How will you measure success of integration?
   - - Key objectives
     - Partnership objectives
     - Your personal measure or objective of success
     - When do you think changes will be shown?
     - When will the population feel the impact?
7. Can you describe if any particular group of people are being targeted through integration?
   - - How will their care be different?
     - How will they be in control of their health and social care?
8. Systems
   - - Are systems in place?
     - Data sharing?
     - Systems fit for purpose?
